# Beak and feather disease virus detected in the endangered Red Goshawk (*Erythrotriorchis radiatus*)

**DOI:** 10.1038/s41598-024-60874-1

**Published:** 2024-05-04

**Authors:** Christopher MacColl, James E. M. Watson, Nicholas P. Leseberg, Richard Seaton, Tridip Das, Shubhagata Das, Shane R. Raidal

**Affiliations:** 1 School of the Environment, The University of Queensland, St Lucia, QLD 4072 Australia; 2 Research and Recovery of Endangered Species Group, The University of Queensland, St Lucia, QLD 4072 Australia; 3 Australian Wildlife Conservancy, P.O. Box 8070, Subiaco East, WA 6008 Australia; 4 School of Agriculture, Environmental and Veterinary Sciences, Faculty of Science, Charles Sturt University, Wagga Wagga, NSW Australia

**Keywords:** Conservation biology, Ecological epidemiology

## Abstract

We report the first detection and prevalence of *Beak and feather disease virus* (BFDV) in Australia’s Red Goshawk (*Erythrotriorchis radiatus*). This is a new host for this pervasive pathogen amongst a growing list of non-psittacine species including birds of prey from the orders *Accipitriformes* (hawks, eagles, kites), *Falconiformes* (falcons and caracas), and *Strigiformes* (owls). The Red Goshawk is the first non-psittacine species listed as Endangered to be diagnosed with BFDV. We report an initial case of infection discovered post-mortem in a dead nestling and subsequent surveillance of birds from across northern Australia. We reveal BFDV prevalence rates in a wild raptor population for the first time, with detections in 25% (n = 7/28) of Red Goshawks sampled. Prevalence appears higher in juveniles compared to adults, although not statistically significant, but is consistent with studies of wild psittacines. BFDV genotypes were associated with the *Loriinae* (lorikeets, budgerigar, and fig parrots), *Cacatuini* (Cockatoos), and *Polytelini* (long-tailed parrots) tribes; species which are preyed upon by Red Goshawks. A positive BFDV status may be associated with lower body mass but small sample sizes precluded robust statistical analysis. We postulate the possible impacts of the virus on Red Goshawks and discuss future research priorities given these preliminary observations.

## Introduction

The Red Goshawk is a taxonomically distinct raptor endemic to the tropics and subtropics of eastern and northern Australia. It is one of Australia’s least detected birds^1^ and is nationally and internationally Endangered^2,3^. It has undergone a significant population decline and range contraction in recent decades with the species now probably extinct across ~ 34% of its breeding range, and declining across an additional ~ 30% of its breeding range^4^. The cause of this widespread and recent decline remains undiagnosed although habitat loss is a likely driver^5^. The species is now considered limited to northern Australia as a breeding resident^4^ having probably bred as far south as northern New South Wales historically^6^. The ecology of the Red Goshawk in these former range areas is little known making diagnosis of the threats driving its widespread extirpation difficult.

Beak and feather disease virus (BFDV) of the genus *Circovirus* is commonly known for infecting parrot species (*psittacines*) in both acute and chronic forms. Chronic cases are characterised by significant feather dystrophy, skin lesions, and beak deformities, whereas the acute form shows diarrhea and feather abnormalities with death often occurring within one-two weeks of developing clinical signs^7^. BFDV is listed as a key threatening process under the *Environmental Protection and Biodiversity Conservation* (EPBC) *Act* of Australia but the Act currently only considers threatened parrots as susceptible to its impacts^7^. It is considered a significant threat to the survival of endangered species with small population sizes and those subject to captive breeding efforts. BFDV has been reported in the majority of Australian psittacine species, with all considered susceptible to infection.

It is emerging that birds of prey are also susceptible to BFDV infection, including members of the *Accipitriformes* (hawks, eagles, kites), *Falconiformes* (falcons and caracas), and *Strigiformes* (owls)^8–10^. This extension to the known host range across a diverse phylogeny of birds known as ‘raptors’^11^ is a recent development but forms part of a growing trend of non-psittacine hosts^8,9,12^. The true taxonomic range and prevalence of BFDV in wild raptor populations is little understood as no field-based studies have been undertaken. Potential impacts on their fitness, breeding success, and survival are likewise unknown.

Verified diagnoses of BFDV in raptors are currently limited to a few acute cases presenting for care or necropsy at veterinary clinics and wildlife hospitals^9,10^. The current list of known BFDV raptor hosts within Australia includes the Brown Goshawk (*Accipiter fasciatus*), Wedge-tailed Eagle (*Aquila audax*), White-bellied Sea-eagle (*Haliaeetus leucogaster*), Peregrine Falcon (*Falco peregrinus*) and Whistling Kite (*Haliastur sphenurus)* amongst the diurnal raptors. BFDV has also been detected in the Powerful Owl (*Ninox strenua*), Southern Boobook (*Ninox boobook*) and Eastern Barn-owl (*Tyto alba*) amongst the nocturnal raptors^8–10^. We now add to this list by reporting BFDV in the Red Goshawk (*Erythrotriorchis radiatus*); the first case of an Endangered non-psittacine species infected with the virus.

Dietary studies from northern Australia confirm it is a bird-eating specialist with avian prey constituting 99.8% of prey items^13^. Of this, parrots are estimated to contribute ~ 51.9% of their dietary biomass. The very closely related Red-collared and Rainbow Lorikeets (*Trichoglossus rubritorquis/moluccanus*) are the most frequently recorded prey species, while larger parrots including the Sulphur-crested Cockatoo (*Cacatua galerita*) are also commonly taken^13^. The two taxonomic groups of which these key prey species belong, the *Loriinae* (lorikeet, budgerigar, and fig parrots) and *Cacatua* (cockatoos), also support the two main genotypes of the BFDV genome.

Clearly there is a significant ecological relationship between parrots and Red Goshawks. However, it is not known if preying predominantly on these known BFDV host species has a deleterious effect on the Red Goshawk’s population. To improve our understanding of how widespread BFDV is in this species, we conducted surveillance of wild birds from across northern Australia. Here, we reveal for the first time, BFDV prevalence rates in a wild raptor population and make preliminary observations on the potential impact to infected hosts. We also discuss possible conservation implications including those associated with the species decline and make future research recommendations given this is the first nationally threatened non-psittacine species diagnosed with BFDV.

## Materials and methods

### Ethics statement

All animal handling procedures and experimental protocols were carried out in accordance with the relevant guidelines and regulations including the Queensland Department of Agriculture and Forestry Animal Ethics Permit (ref: CA 2018/04/1183 and CA 2020-11-1437), the Queensland Department of Environment, Science, and Innovation Scientific Purposes Permit (ref: WA0008989), the Northern Territory Parks and Wildlife Commission Permit to Interfere with Wildlife (ref: 65,181), the Western Australian Department of Biodiversity, Conservation, and Attractions Authorisation to Take or Disturb Threatened Species (ref: TFA 2019-0079-2), and the Australian Department of the Environment and Energy Part 13 Permit (ref: E2019-0163). This study is also reported in accordance with the ARRIVE guidelines and conforms to its principles.

### Data collection

A total of 28 biological samples were collected from individual Red Goshawks across northern Australia including Queensland (n = 22), the Northern Territory (n = 5), and Western Australia (n = 1) between 2015 and 2022. Four methods were used to obtain these samples: (1) feathers plucked from live birds (i.e. body coverts) during capture (n = 19); (2) feathers plucked from dead birds found beneath nests (n = 2); (3) feathers found on the ground beneath nests (i.e. flight feathers) following moult by breeding adults (n = 6); and (4) liver samples collected from dead birds found beneath nests (n = 1). Red Goshawk were captured around nest sites via a remotely controlled 8 ft bow-net (Northwoods Falconry, Olympia, WA, USA) using a live lure bird as the attractant.

Active surveillance of BFDV in Red Goshawk commenced after its initial discovery in 2021 following test results obtained from a dead nestling found beneath a nest in 2019. However, feather samples collected since 2015 were able to be used retrospectively in our BFDV screening. All feathers were kept in separate re-sealable bags to avoid cross contamination between samples and stored at either at room temperature (n = 5), refrigerated (n = 23), or frozen (n = 1).

Morphological data were collected from captured individuals including wing length (mm), tail length (mm), culmen length (mm), tarsus length (mm), and body mass (g). Due to inherent size differences between the adult and juvenile age classes, our analysis of morphological traits and BFDV status were limited to the juvenile age cohort to ensure our results were comparable between individuals. Furthermore, there was insufficient data to compare potential morphology differences between infected and uninfected adults as there were only two confirmed cases of infection.

Age and sex were assigned where known through in-hand measurements or visual observation by experienced observers. Moulted feathers found on the ground could only be assigned to the adult age class with sex unable to be determined. Where multiple feather samples were collected at the same nest both on ground and from live birds, the latter sample was used in our analysis to avoid possible duplicate counts of the same individuals. Two samples were collected from nestlings, but these data were grouped with fledglings under the ‘juvenile’ age cohort for our analysis.

### Lab analysis

The mean sampling time between sample collection and lab analysis was 470 days (SD = 567). BFDV is resilient and able to persist in the environment for long periods of time^8^. BFDV DNA was extracted from feather pulp and blood samples using established techniques and screened for BFDV by an established PCR protocol that targets the 717-bp section of the BFDV^10,12^. Purified amplicons were sequenced with PCR and internal primers by the Australian Genome Research Facility Ltd (Sydney) using an AB 3730xl unit (Applied Biosystems). For each amplicon sequences were obtained at least twice in each direction for each isolate to ensure the validity of the data. Amplicon sequencing using material contained within feather pulp also avoids using external feather surfaces during testing which may be prone to cross-contamination. For example, the handling of infected prey by Red Goshawks including plucking feathers could lead to false positive results if viral material is shed onto their own feather surfaces during consumption. The sequences were trimmed for primers, aligned to construct contigs using a minimum overlap of 35 bp and a minimum match percentage of 95%, and construction of full genome and partial sequences were carried out in Geneious Pro and BioEdit Sequence Alignment Editor (version 7.1.6.0). These were aligned with publicly available BFDV genomes from different regions of Australia using Geneious with MAFFT v7.017 and using the G-INS-i (gap open penalty 1.53; offset value 0.123) alignment algorithm.

### Data analysis

Detections and non-detections of BFDV were used to calculate prevalence rates and 95% confidence intervals according to age, sex, and region. Fisher’s exact tests were used to determine if there was a significant association between these factors and BFDV prevalence rates. Sample collection points were used to plot all known detections geographically in order to spatially assess the virus’ known extent of occurrence in Red Goshawk. Morphological traits were plotted according to BFDV status to assess for discrepancies between infected and non-infected individuals. Males and females were separated given their large, non-overlapping size difference. All analyses and mapping were conducted in R^14^ using the ggplot2^15^, sf^16^, and tmap^17^ packages.

## Results

**Figure 1:**
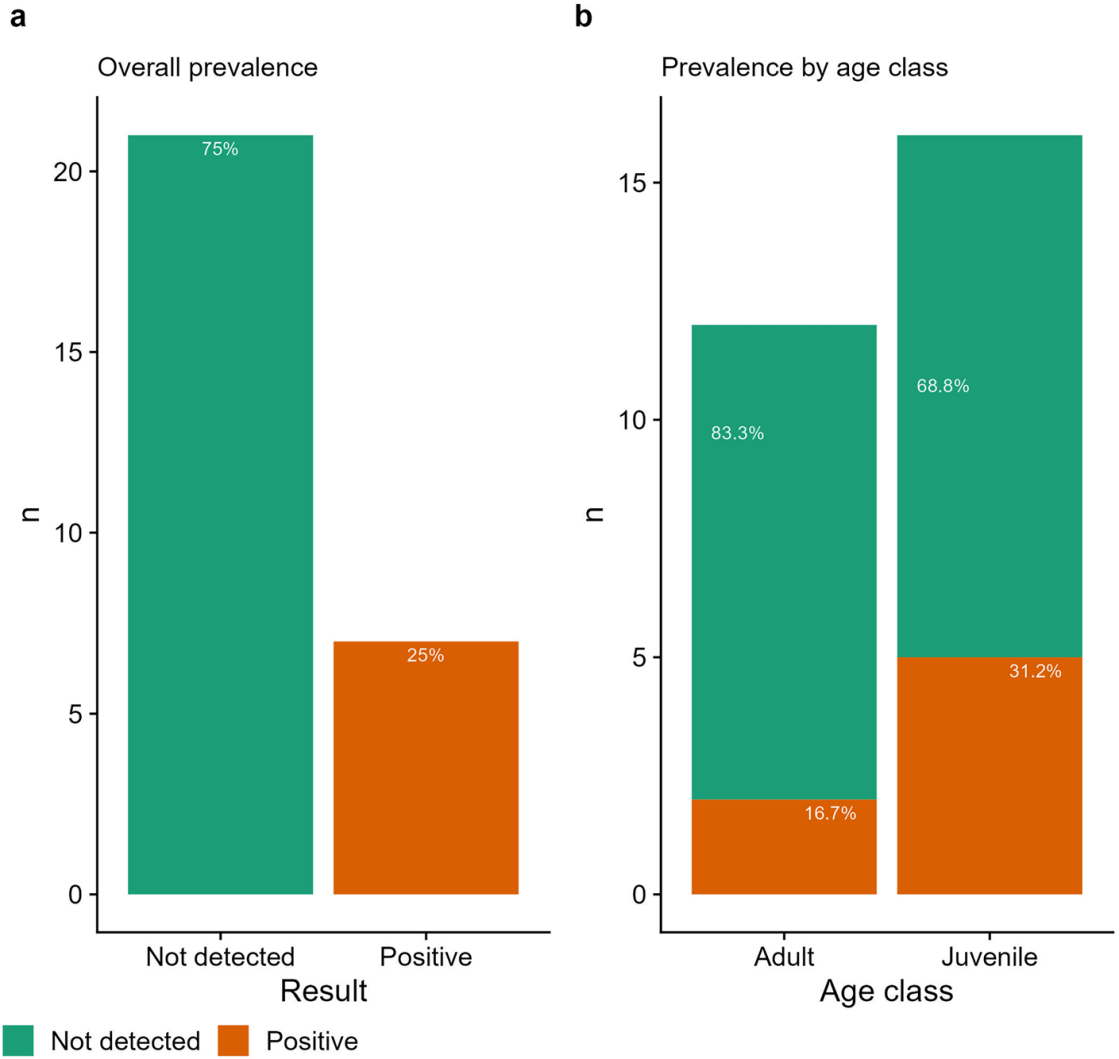
Results of Beak and Feather Disease Virus screening in Red Goshawks. (**a**) Overall prevalence across all individuals. (**b**) Prevalence by age class between adults and juveniles.

BFDV was detected in seven individuals and not detected in 21 individuals, placing the overall prevalence rate of our sampled population at 25% [n = 7/28, 95% CI 12.7–43.4] (Fig. 1a). Higher prevalence was recorded in juveniles at 31.2% [n = 5/16, 95% CI 0.14–0.56] compared to 16.7% in adults [n = 2/12, 95% CI 0.05–0.45]; however, a Fisher’s exact test revealed no significant association between BFDV prevalence and age class (*p* = 0.66) (Fig. 1b). Similarly, no statistical significance was found between BFDV prevalence and sex (*p* = 1) with males [33.3%, n = 2/6, 95% CI 0.10–0.70] and females [28.6%, n = 4/14, 95% CI 0.12–0.55] showing similar rates of infection.

**Figure 2:**
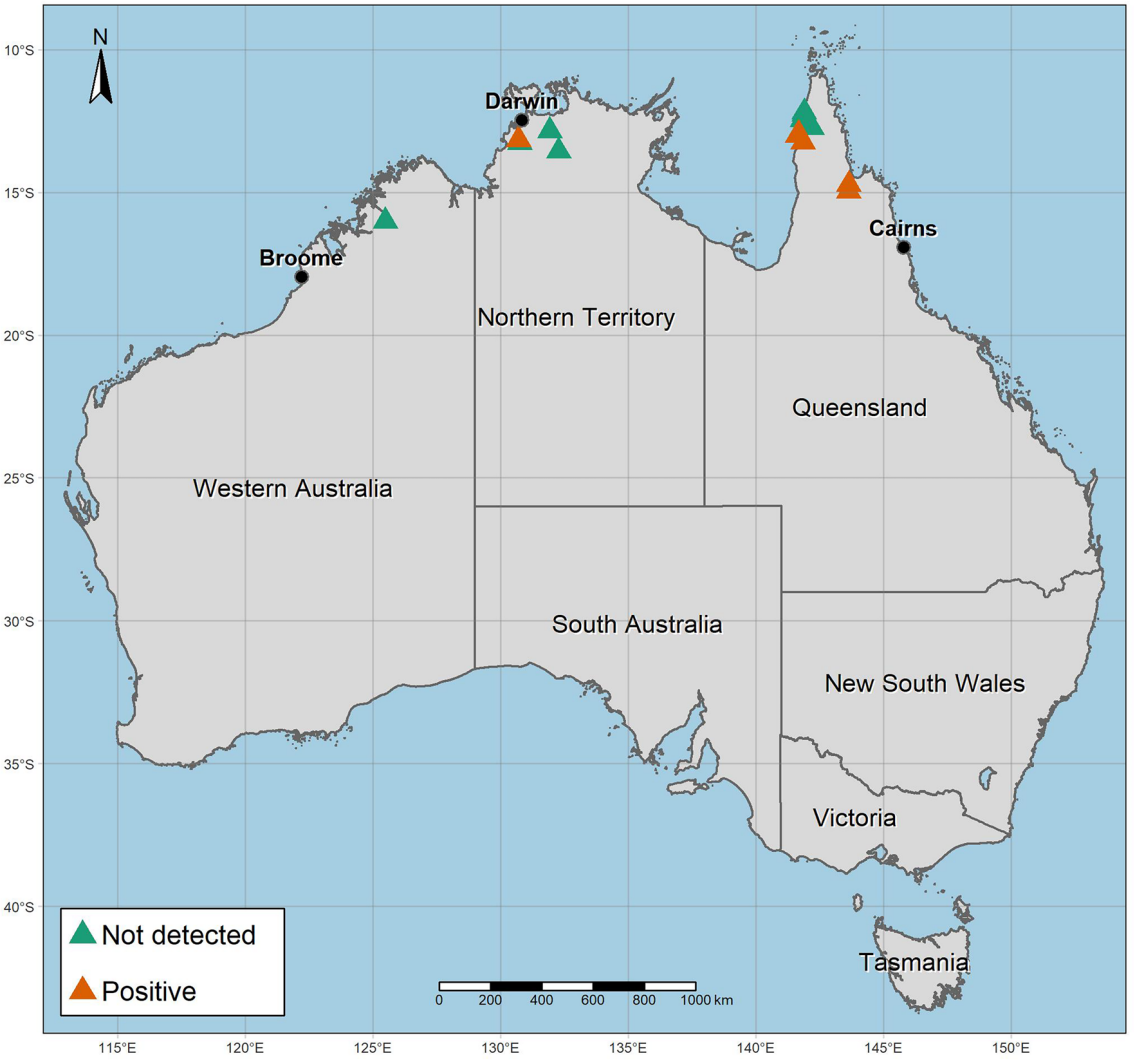
Sampling locations and results of Beak and Feather Disease Virus screening in Red Goshawks across northern Australia including the states of Queensland, the Northern Territory, and Western Australia.

Regionally, six individuals tested positive for BFDV in Queensland for a prevalence rate of 27.3% [n = 6/22, 95% CI 0.13–0.48]. Just one individual tested positive for BFDV in the Northern Territory for a prevalence rate of 20% [n = 1/5, 95% CI 0.04–0.62], albeit based on very limited data. No detections were made in Western Australia although only one sample was obtained for analysis. The sampling locations and positive results illustrate the known spatial extent of the virus in Red Goshawks which at least incorporates the northern tropical savanna areas of Queensland and the Northern Territory (Fig. 2).

**Figure 3:**
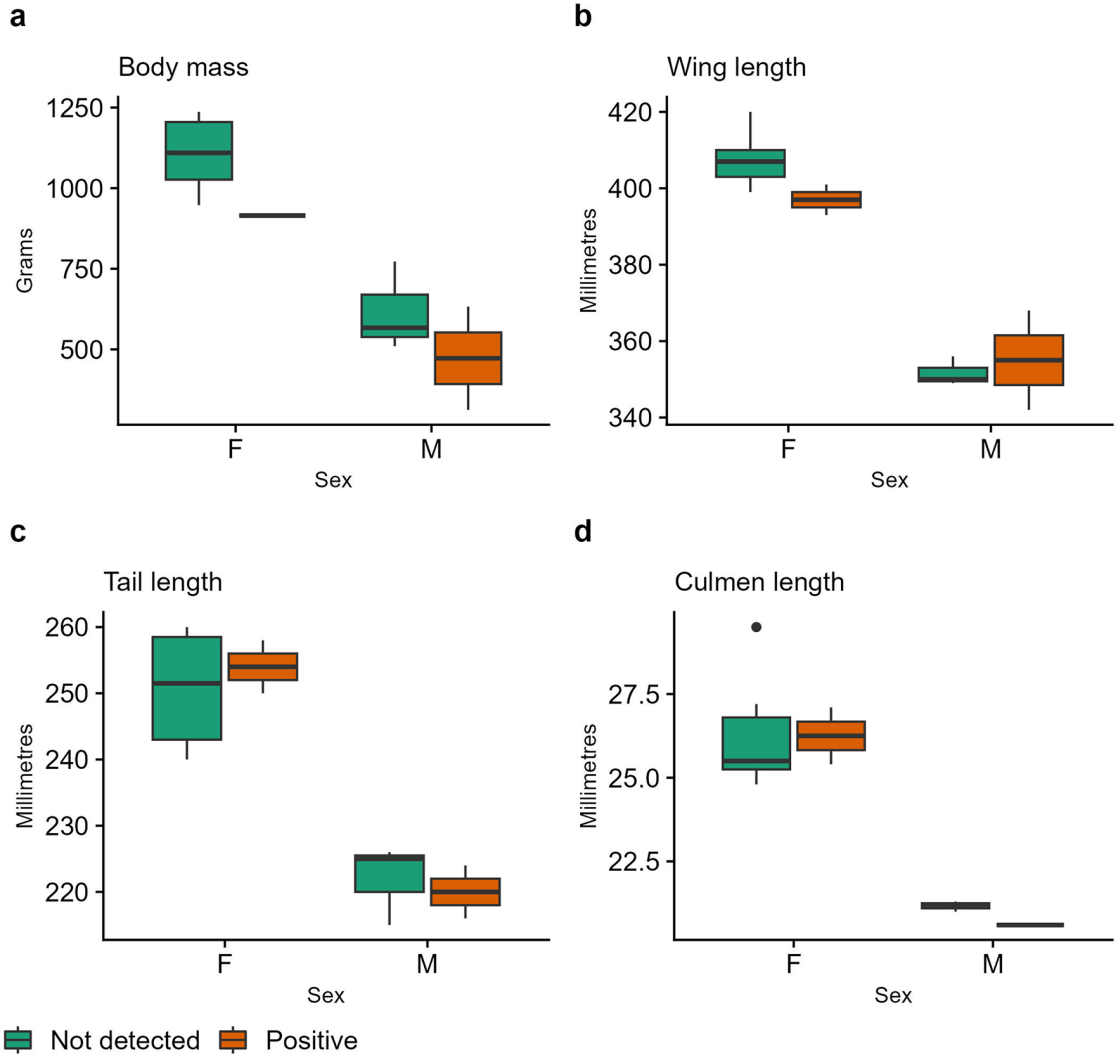
The distribution of morphological data between juvenile male and female Red Goshawks according to their BFDV status. (**a**) Body mass. (**b**) Wing chord length. (**c**) Tail length. (**d**) Culmen length.

Sample sizes were too low to allow robust statistical comparison between positive and negative cases across morphological measurements in juveniles. These data indicate body mass is lower in hosts (n = 3) compared to non-hosts (n = 8), but larger sample sizes are needed to test this conclusively, particularly given the need to separate results by sex (Fig. 3a). The distribution of wing, tail, and culmen length did not indicate any discernible difference between positive and negative cases but these data are likewise insufficient to allow valid statistical comparison between groups (Fig. 3b–d).

Phylogenetic analysis revealed that the BFDV genotypes from Red Goshawks are incorporated into four distinctly separated phylogenetic clades (Table 1; Supplementary Fig. 1). Samples RGG31 and RGG32 had a cockatoo specific BFDV genotype which occurs within a clade comprised of spatially indiscriminate host generalist BFDV genotypes from *Cacatuini*. Sample RGG35 had a BFDV genotype closely related to the *Polytelini* tribe, which forms a strongly supported monophyletic clade. The Red-winged Parrot (*Aprosmictus erythropterus*), a known though infrequent prey species of Red Goshawk, belongs to the tribe *Polytelini*. Samples RGG08, RGG30, and RGG38 all clustered together within a strongly supported clade dominated by host tribe *Lorini*. Phylogenetic resolution for BFDV genotype in sample RGG23 was not clear as it branched out from a clade formed by BFDV genotypes in host tribe *Pezoporini*. However, there was weak bootstrap support (< 50%) between clade *Pezoporini* and *Cacatuini*. Given that *Pezoporini* includes small grass parrots (*Neophema* sp.) and ground parrots (*Pezoporus* sp.) from outside our study region predominantly in southern Australia, it is likely that the BFDV genotype in RGG23 indeed belongs to *Cacatuini*.

**Table 1:** Positive cases of Beak and Feather Disease Virus in Red Goshawks including sampling, demographic, and genetic information.

Sample ID	Sample type	Age class	Sex	Genotype
RGG08	Feather (live sampling)	Adult	Female	*Lorini*
RGG23	Feather (live sampling)	Adult	Female	*Pezoporini*
RGG30	Feather (live sampling)	Juvenile	Male	*Lorini*
RGG31	Feather (live sampling)	Juvenile	Male	*Cacatuini*
RGG32	Feather (live sampling)	Juvenile	Female	*Cacatuini*
RGG35	Feather (collected on ground)	Juvenile	Female	*Polytelini*
RGG38	Liver	Juvenile	Unknown	*Lorini*

## Discussion

This research presents the first known surveillance of BFDV in a wild raptor population, and documents the first known case, and possible impacts, of an *Endangered* non-psittacine species infected with the virus. However, caution is needed in assessing the potential threat this virus poses to Red Goshawk as it has not clearly been established that infection leads to chronic disease in the adult and fledgling-age birds we mostly sampled. Hosts may carry the virus but ultimately show no clinical signs^8^ and our study did not include follow up pathological monitoring of sampled birds except by visual observation of tagged breeders whom return to known nesting locations (i.e. compared to widely dispersed juveniles). Susceptibility to the virus is age-related and it is on this basis that we postulate the potential impacts an infected population may be exposed to based on our results and findings from better studied species.

### Potential impacts

An overall prevalence rate of 25% (95% CI 0.13–0.43) in Red Goshawks is comparable with most studies of wild psittacine host populations including the New Caledonian Rainbow Lorikeet (*Trichoglossus haematodus deplanchii*) at 25%^18^, Sulphur-crested Cockatoo at 10–20%^19^, Crimson Rosella (*Platycercus elegans*) at 41.8%, Blue-winged Parrot (*Neophema chrysostoma)* at 11.8%, and Galah (*Eolophus roseicapilla*) at 8.8%^20^. However, higher rates have been reported in some species or populations, including the Little Corella (*Cacatua sanguinea*) at 100%, Long-billed corella (*Cacatua tenuirostris*) at 56.7%, and Sulphur-crested Cockatoo at 92.3%^21^. Limited studies have assessed prevalence rates according to age class but the available evidence suggests younger birds are more likely to become infected^8^. Controlled studies revealed infection was most prolific amongst nestling age Budgerigars (*Melopsittacus undulatus*) and Galahs^22^. Mortality appears most likely to occur at the nestling or fledgling stage, with these younger birds often succumbing to acute forms of the disease^8^. The prevalence rates we have observed amongst fledged Red Goshawk suggests there could be cases of undetected mortality occurring inside the nest given this age-related susceptibility^8,22^. Increased nestling mortality could reduce nest productivity and recruitment into the adult population. However, recent fledgling rates in the Northern Territory appear consistent with historical baselines^13^ including a longitudinal study on the Tiwi Islands^23^ but these trends need to be investigated further. In order to adequately assess for these impacts, more comprehensive breeding data are needed, and larger sample sizes of BFDV prevalence. In-nest monitoring is also required if disease exposure and potential breeding impacts are to be investigated at all stages of reproduction including the periods of highest vulnerability.

BFDV causes characteristic beak and feather abnormalities including bill elongation and persistent feather loss yet there were no discernible differences between juvenile hosts and non-hosts across wing, tail, or culmen measurements based on our limited data. There were also no obvious clinical signs of infection in monitored individuals, but this is also consistent with studies of wild cacatuids in southeastern Australia^21^. The potential difference in body mass distribution is interesting and worthy of further investigation if sufficient sample sizes can be collected across age and sex classes. Lower body mass may indicate a physiological impact which leads to reduced fitness and lower survival probability even if the young are able to successfully fledge from the nest and disperse from the natal territory. For example, studies of wild cockatoos found the 2–3-year-old age cohort had the most significant feather loss^8^. Chronic immunosuppressed birds often succumb to secondary causes such as other disease problems or predation^21,24,25^. In order for the disease to manifest, one or two moult cycles may be required, but most birds that succumb to BFDV are < 2 years old^8^. As such, further work is also needed to assess for any delayed impact on the survival of young birds that fledge and reach independence yet were infected within the nest. Dead birds found beneath nests also represent highly valuable specimens for pathological investigation and should be collected, particularly if found prior to advanced decomposition where viable tissue and blood samples can be obtained.

No discernible difference in rates of infection between males and females is also consistent with studies of wild parrots whereby species rather than sex is a predictor of BFDV infection^20^. The susceptibility of nestlings would suggest brooding adult females are similarly exposed to the virus and indeed our only confirmed cases of adult infection belong to adult females. However, adult males are poorly represented in our data set, at least amongst birds directly sampled where sex was known (i.e. compared to moulted feathers found on the ground where sex is unknown), so sex related differences at the adult age class cannot be ruled out. However, adult males provision the female and young for most of the breeding season so if transmission occurs through the handling and consumption of infected prey then males would be similarly exposed to infection.

### Preliminary observations of known cases

The initial discovery of BFDV in this species came from an approximately one week old nestling found deceased beneath a nest in Queensland during the 2019 breeding season. While BFDV cannot be confirmed as the cause of this mortality, it should be considered a strong possibility, particularly given that nestling age is the period of highest susceptibility to the virus^8,22^. The following season resulted in another dead nestling of approximately four weeks old being found beneath the same nest, although that nestling was unable to be retrieved for testing.

Fledglings captured for tracking-based survival studies over the 2021–2022 breeding seasons have provided a mixed sample of both BFDV positive and negative cases. It is currently unclear if a positive BFDV status is a predictor of first-year mortality as this study is still ongoing. Mortality is also typically high in first-year raptors, particularly bird-eaters^26^, so greater sample sizes would be needed to assess the influence of BFDV infection as a predictor of survivorship.

The two adult cases are both breeding females subject to tracking and breeding studies. Their survival has apparently not been impacted by their positive BFDV status, as both birds continue to survive after more than two years of tracking. While infected, four of the five breeding attempts involving these two females have produced fledged young. The one breeding failure apparently involved predation of the nestling, which was found beneath the nest, but did not test positive for BFDV. Their ability to thrive over multiple seasons suggests they are carriers of this virus and remain asymptomatic. It would be expected that younger birds are more likely to display clinical signs of infection particularly during feather development. However, whilst this has been shown in psittacine species^8,22^, it remains a gap in our understanding of how BFDV may impact the Red Goshawks population and other non-psittacine predatory species including raptors.

### Red Goshawk decline

BFDV is endemic and has persisted in Australia for millions of years^8^. Such a deep evolutionary history suggests that native species have been able to co-exist with the virus. However, anthropomorphic changes to the landscape, particularly due to habitat alteration over the last 230 years since European colonisation, may have changed the abundance of psittacine host populations, and potentially altered the virus’ prevalence and extent along with it. For example, Sulphur-crested Cockatoo, Little Corella, and Rainbow Lorikeet populations have increased across urban and rural areas of greater Sydney, Melbourne, and Brisbane^28–31^. Given their hollow-nesting requirements, and with these resources more limited in the landscape due to clearing, it is likely that transmission has increased over time due to inter-seasonal nest hollow sharing^8^. Whilst longitudinal studies of BFDV prevalence rates over time do not exist, current estimates are 5–20% in the juvenile population^19,32^.

The potential for BFDV to have contributed to the decline of the Red Goshawk is circumstantial. There are no data which can be used retrospectively as no nests were monitored to detect declines due to disease, and no viable biological material ever collected from the regions of eastern Australia from which the Red Goshawk has apparently disappeared. Red Goshawks clearly share a close ecological relationship with parrots given they are a dietary staple, at least in northern Australia^13^. Dietary information from the historical parts of their range throughout eastern Australia are lacking, but early records do document parrots being preyed upon. One of the earliest specimens, and the most southerly recorded adult, was a male observed hunting rainbow lorikeets in the Clarence Valley NSW in 1866^33^, and there are other early records of the species preying on psittacines^34–36^. Whilst a dependence on psittacines as a primary food resource historically cannot be established from such little information, it is known that psittacine populations capable of hosting BFDV achieve their highest densities in some of these regions^37^. Based on this distribution of potential host species, interactions appear highest throughout eastern and south-eastern Australia^37^. Therefore, we posit that a spatial relationship exists between concentrations of BFDV host species and Red Goshawk extirpation, although this is again only correlative.

### Future research recommendations

More detailed information on the outcomes of nesting attempts is necessary to draw robust conclusions about the potential impacts of BFDV on the Red Goshawk population. Tracking the number of eggs produced, eggs hatched, nestling survival, and fledging success is required to better detect any trends (e.g. nestling survival) that could be attributed to the effects of BFDV over time. To determine whether there is any delayed physiological impact to juveniles (e.g. feather loss) which manage to fledge and disperse from their natal territories but are infected with the virus may require remote tracking for follow up visual or pathological monitoring. Ongoing collection of moulted feathers and retrieval of any remains surrounding nests is also recommended in order to continue building a database on BFDV prevalence rates and possible associated mortalities. The live capture of birds for direct sampling, particularly amongst juveniles which appear to have higher prevalence rates, will also add valuable data to continue investigating whether infected birds exhibit physiological impacts such as lower body mass. Comparison of BFDV prevalence in wild psittacine populations across extant and extirpated areas of the Red Goshawks range (e.g. northern Australia vs. south-eastern Australia) may provide further insight into possible exposure rates to the virus and whether it varies geographically.

## Conclusion

BFDV is a pervasive pathogen capable of infecting a broad range of hosts, now extending to Endangered non-psittacine species such as the Red Goshawk. This species is undergoing a severe population decline and range loss. A pathogen such as BFDV may represent a pervasive threat to the species, but further work is needed to better understand its potential impact. Prevalence rates are quite high, comparable with psittacine hosts, with juveniles potentially more infected albeit based on limited sample sizes. Whether this has a deleterious effect on the species’ breeding rates or survival is unknown and worthy of further investigation as the results we present here are preliminary.

### Supplementary Information

Supplementary Information 1.

Supplementary Figure 1.

## Data Availability

The data that support the main findings of this study are contained within the manuscript or supplementary material.
