# Supplementary material for: Beak and feather disease virus detected in the endangered Red Goshawk (Erythrotriorchis radiatus)
Source: Sci Rep. 2024 May 4;14:10263. doi: 10.1038/s41598-024-60874-1 (PMC11069563; doi:10.1038/s41598-024-60874-1)
Supplement: Supplementary file 2 — Supplementary Figure 1. [file 41598_2024_60874_MOESM2_ESM.docx]

**Supplementary information**


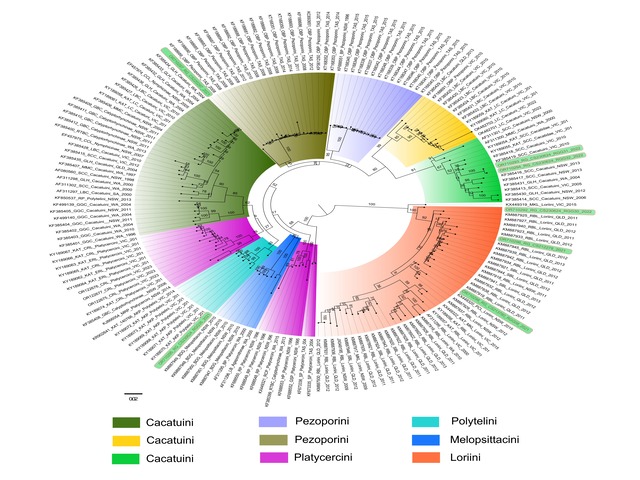


Supplementary Figure 1. Maximum likelihood (ML) tree inferring phylogenetic relationship of BFDV in red goshawk in context of extant BFDV genetic clusters in Australia. Labels at branch tips refer to GenBank accession numbers with country name, species abbreviation, taxonomic tribe of host and year of sampling. Clade posterior probability values are shown at tree nodes. Labels at branch tips refer to GenBank accession numbers, host taxonomic tribes, country and year of collection. Taxa in Apple green background shading represents the BFDV sequences generated in this study from natural infection in red goshawk while colour-coded in phylogenetic clade highlights known BFDV genotypic clusters circulating in Australian reservoirs.
